# Patient-Related Progression of Steeper Sternal Wire Angles: A Case Report

**DOI:** 10.7759/cureus.71324

**Published:** 2024-10-12

**Authors:** Tomohiro Nakajima, Tsuyoshi Shibata, Yu Iwashiro, Yutaka Iba, Nobuyoshi Kawaharada

**Affiliations:** 1 Cardiovascular Surgery, Sapporo Medical University, Sapporo, JPN

**Keywords:** chest x-p, computed tomography, remove, steep, sternal wire

## Abstract

A 78-year-old female presented with a history of left atrial myxoma resection 12 years before presentation. The initial surgery involved a median sternotomy and cardiopulmonary bypass for tumor excision. Sternal closure was achieved using six titanium wires, with the lowermost wire noted to be slightly elevated from the sternum immediately post-operation.

The patient, an active individual who regularly practiced yoga, including frequent prone positions, was discharged from follow-up two years postoperatively without complications. However, 12 years after the initial surgery, the patient experienced pain at the lower end of the median sternotomy site, prompting her first visit to our outpatient clinic within a decade.

Physical examination revealed palpable subcutaneous protrusion of the lowermost sternal wire with visible skin discoloration. Although no evidence indicated wire penetrating the skin, wire removal was deemed necessary. Comparison with previous lateral chest radiographs demonstrated a progressive increase in wire angulation over time.

The patient was admitted and the protruding wire was removed under local anesthesia. Her postoperative course was uneventful, leading to discharge on the sixth day after the procedure.

## Introduction

Sternal wire cerclage remains a standard technique for sternal closure following median sternotomy, with titanium wires being a common choice due to their biocompatibility and strength. However, sternal wire complications, including wire fracture, migration, and protrusion, can occur years after the initial surgery, causing significant discomfort and increasing the potential risk of infection in patients.

The mechanism underlying sternal wire protrusion is multifactorial. Khouzam et al. proposed that wire protrusion could result from a combination of mechanical stress, tissue remodeling, and patient-specific factors. The authors noted that repetitive movements of the chest wall, particularly in active patients, could lead to gradual wire displacement over time [1].

Furthermore, Tomizawa et al. suggested that bone resorption around the wires, which is potentially exacerbated by osteoporosis in older patients, can contribute to wire loosening and subsequent protrusion [2]. This process may be accelerated in patients engaging in activities involving frequent changes in chest wall pressure, such as yoga, or other exercises that require prone positioning.

Sternal wire complications often present as localized pain, palpable subcutaneous protrusions, or visible skin changes at the sternal closure site. Although rare, the delayed presentation of these complications can occur years after the initial surgery, as demonstrated by Rupprecht et al., who reported a case of wire protrusion 15 years after sternotomy [3].

## Case presentation

**Figure 1:**
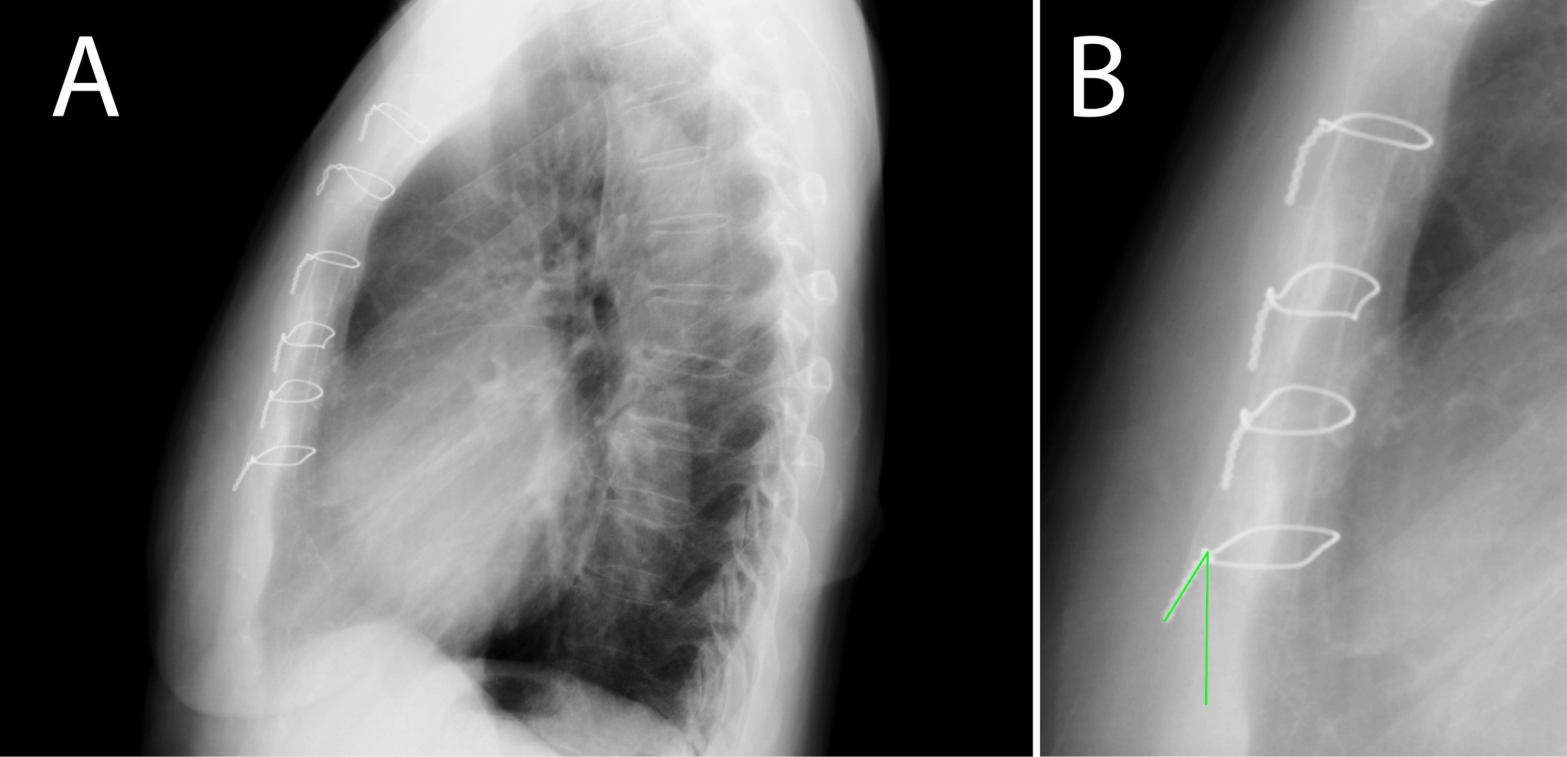
Chest X-p after the first operation (A) Lateral chest X-p reveals the sixth wire standing up. (B) The angle between the vertical line is 31.4° (green line). Chest X-p: chest X-ray photograph

A 78-year-old female presented with a history of left atrial myxoma resection 12 years before presentation. The initial surgery involved a median sternotomy and cardiopulmonary bypass for tumor excision. Sternal closure was achieved using six titanium wires. Notably, the lowermost wire was slightly elevated from the sternum immediately after the surgery (Figures 1A, 1B) at an angle of 31.4° from the vertical plane.

**Figure 2:**
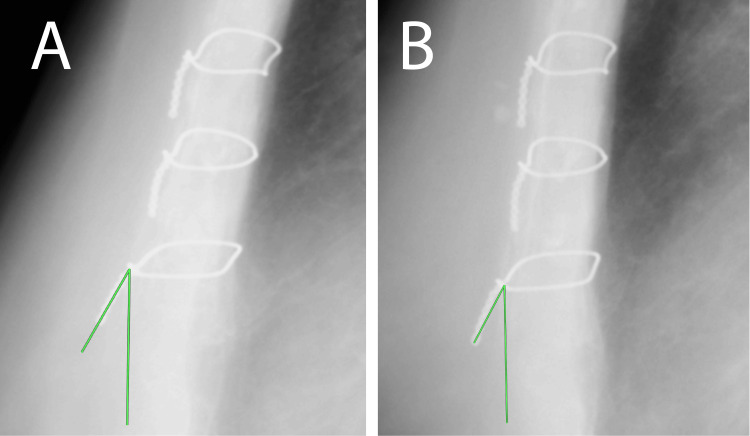
Chest X-p after two and six years (A) Two years post-procedure. The angle between the vertical line is 27.2° (green line). (B) Six years following the procedure. The angle between the vertical line is 28.8° (green line). Chest X-p: chest X-ray photograph

The patient was discharged from follow-up two years post-surgery without complications (Figure 2A). A chest radiograph acquired for an unrelated reason six years post-operation displayed no significant change in wire angulation (Figure 2B).

The patient, an active individual who regularly practiced yoga, frequently engaged in prone positioning during their routine exercises. The patient said that she had started to feel pain in the wires in her breastbone after starting yoga. Twelve years after the initial surgery, the patient experienced pain at the lower end of the median sternotomy site, prompting her to visit our outpatient clinic.

**Figure 3:**
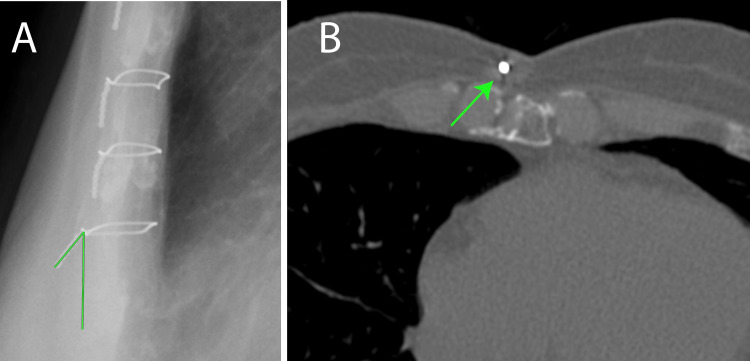
Chest X-p after 14 years (A) Lateral chest X-p demonstrates the sixth wire standing up. The angle between the vertical line is 39.0° (green line). (B) Computed tomography displays no evidence of wire penetration through the skin (green arrow). Chest X-p: chest X-ray photograph

Physical examination revealed a palpable subcutaneous protrusion of the lowermost sternal wire with visible skin discoloration (Figure 3A). Computed tomography revealed no evidence of wire penetration through the skin (Figure 3B); however, a comparison with previous lateral chest radiographs demonstrated a progressive increase in wire angulation over time.

**Figure 4:**
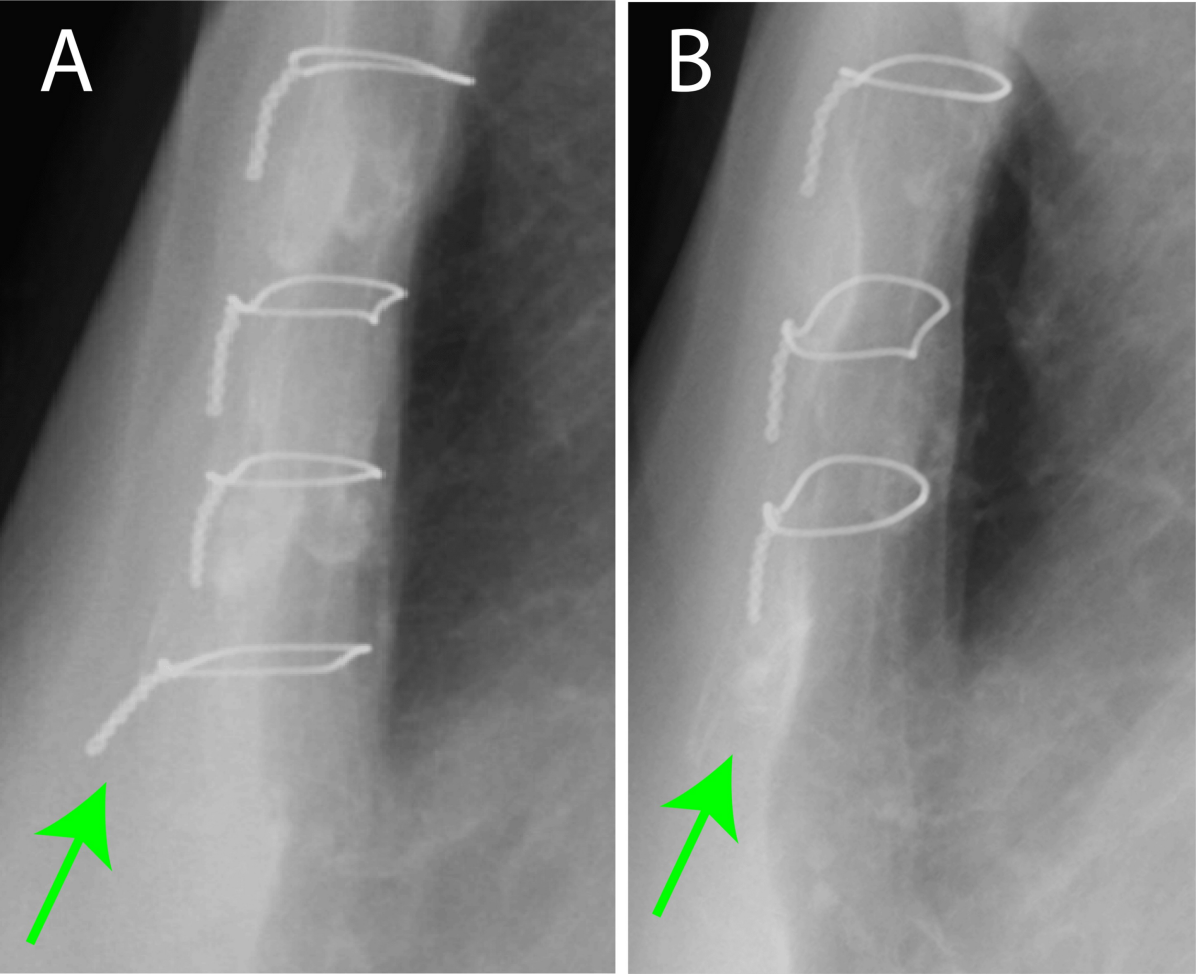
Chest X-p before and after wire removal (A) Before wire removal. Lateral chest X-p reveals the sixth wire was standing up. The angle between the vertical line is 39.0° (green arrow). (B) After wire removal. The sixth wire is removed (green arrow). Chest X-p: chest X-ray photograph

The protruding wire was removed under local anesthesia following the admission of the patient. The patient’s postoperative course was uneventful (Figure 4), leading to discharge on the sixth postoperative day.

## Discussion

Sternal wire closure following median sternotomy for cardiac surgery is a universal technique [4]. It plays a crucial role in preventing sternal instability, reducing postoperative pain, and facilitating sternal union. In thin patients, sternal wires may be palpable subcutaneously [5]. Rarely, chronic compression can lead to wire penetration through the skin, necessitating intervention due to infection risk [6]. In recent years, new sternum closure devices and methods have been reported [7,8].

In this case, the patient remained asymptomatic for six years post-surgery. The wire protrusion appears to have been precipitated by the patient's habitual practice of yoga, particularly exercises involving prone positioning. It is thought that aging has reduced the amount of fatty tissue in front of the sternum, which has brought the sternal wires closer to the skin and made them more susceptible to external forces. Early intervention, prior to wire penetration through the skin, allowed for a brief hospital stay and uncomplicated recovery.

Notably, the initial surgery left the wire ends relatively long and slightly elevated from the sternum. Had the wires been cut shorter and positioned at a smaller angle to the sternum, the need for wire removal might have been avoided.

This indicates that postoperative activity guidelines should be stricter for certain patient populations, and clinicians may need to consider alternative closure methods, such as rigid fixation systems, for individuals with active lifestyles to minimize the risk of wire migration.

Additionally, alternative closure techniques should be considered for high-risk groups, including thin patients, where traditional wire cerclage might not be ideal due to subcutaneous wire palpability and potential skin penetration.

## Conclusions

We report a case of sternal wire protrusion that required removal 14 years after median sternotomy for cardiac surgery. The protrusion was likely attributed to a combination of factors: initially long and slightly elevated wire ends from the primary surgery, which were exacerbated by the patient's regular yoga practice in the prone position. This case underscores the importance of proper wire management during sternal closure, specifically cutting the short wire ends and ensuring firm compression of the sternum to minimize the risk of long-term complications.

This experience highlights the need for meticulous attention to detail during sternal closure and emphasizes how patient activities can influence long-term outcomes. The study also demonstrated that complications from sternal wires can occur many years after the initial surgery, suggesting the importance of patient education and long-term follow-up for high-risk cases.
